# Risperidone Controlled Release Microspheres Based on Poly(Lactic Acid)-Poly(Propylene Adipate) Novel Polymer Blends Appropriate for Long Acting Injectable Formulations

**DOI:** 10.3390/pharmaceutics10030130

**Published:** 2018-08-13

**Authors:** Stavroula Nanaki, Panagiotis Barmpalexis, Alexandros Iatrou, Evi Christodoulou, Margaritis Kostoglou, Dimitrios N. Bikiaris

**Affiliations:** 1 Laboratory of Polymer Chemistry and Technology, Department of Chemistry, Aristotle University of Thessaloniki, 54124 Thessaloniki, Greece; sgnanaki@chem.auth.gr (S.N.); iatroualexandros@hotmail.gr (A.I.); evicius@gmail.com (E.C.); 2 Department of Pharmaceutical Technology, School of Pharmacy, Aristotle University of Thessaloniki, 54124 Thessaloniki, Greece; pbarmp@pharm.auth.gr; 3 Laboratory of General and Inorganic Chemical Technology, Aristotle University of Thessaloniki, 54124 Thessaloniki, Greece; kostoglu@chem.auth.gr

**Keywords:** poly(lactic acid), poly(propylene adipate), polyester blends, risperidone, controlled release, long-acting injectable, microspheres

## Abstract

The present study evaluates the preparation of risperidone controlled release microspheres as appropriate long-acting injectable formulations based on a series of novel biodegradable and biocompatible poly(lactic acid)–poly(propylene adipate) (PLA/PPAd) polymer blends. Initially, PPAd was synthesized using a two-stage melt polycondensation method (esterification and polycondensation) and characterized by 1H-NMR, differential scanning calorimetry (DSC), and powder X-ray diffraction (XRD) analyses. DSC and XRD results for PLA/PPAd blends (prepared by the solvent evaporation method) showed that these are immiscible, while enzymatic hydrolysis studies performed at 37 °C showed increased mass loss for PPAd compared to PLA. Risperidone-polyester microparticles prepared by the oil–water emulsification/solvent evaporation method showed smooth spherical surface with particle sizes from 1 to 15 μm. DSC, XRD, and Fourier-transformed infrared (FTIR) analyses showed that the active pharmaceutical ingredient (API) was dispersed in the amorphous phase within the polymer matrices, whereas in vitro drug release studies showed risperidone controlled release rates in all PLA/PPAd blend formulations. Finally, statistical moment analysis showed that polyester hydrolysis had a major impact on API release kinetics, while in PLA/PPAd blends with high PLA content, drug release was mainly controlled by diffusion.

## 1. Introduction

Drug parenteral administration is the main choice of treatment especially in cases where the active pharmaceutical ingredient (API) exhibits high first-pass metabolism, is highly unstable in the gastrointestinal physiological conditions or shows a narrow therapeutic index. Despite the aforementioned advantages several limitations regarding patient compliance, frequent (every-day) use of injections and needle-phobia, possess an important drawback in treatment acceptance. In order to overcome these, the reduction of the total number of injections through the use of properly designed depot controlled release parenteral formulations has shown to be truly advantageous, especially in cases where long-term treatment is needed [1,2,3,4]. Research on such drug delivery systems has been growing increasingly in the last decades due to the several advantages that they possess, including better patient compliance through lower dosing frequency, consistent drug blood levels, reduced adverse effects, avoidance of first-pass metabolism etc. [5]. Especially in the case of schizophrenia, important new formulation approaches in the area of drug delivery have been introduced since the first conventional depot formulation for the delivery of an antipsychotic API was introduced [6].

Several bio-degradable polymeric materials have been tested as suitable carriers for the preparation of parenteral depot formulations, including natural polymers, such as albumin starch, dextran, gelatin, and hemoglobin, and synthetic polymers, such as polyacrylamides, polyamino acid, polyurethane, and aliphatic polyesters, such as poly(lactic acid) (PLA), poly(glycolic acid) (PGA), poly(lactide-*co*-glycolide) (PLGA), poly(ε-caprolactone) (PCL), etc. [7,8,9,10,11,12]. The major advantages of using biodegradable polymeric materials is that they show low toxicity, do not require surgical removal after drug exhaustion, and can be modified in such a way as to achieve a desirable drug delivery profile [13]. Generally, biomaterials prepared from aliphatic polyesters, such as copolymers of lactic and glycolic acids (PLA, PGA, and PLGA) are widely used in pharmaceutical formulations [14,15,16,17,18,19]. Aliphatic polyester based particulate systems, such as microparticles and nanoparticles, have shown increased capability for delivering a variety of APIs, including peptides [19], proteins [20], vaccines [18], and antipsychotic drugs [15]. Drug release from such systems is governed by simultaneous drug diffusion and polymer erosion [20,21].

Regarding antipsychotic API depot parenteral treatment, a risperidone controlled release injectable formulation (in the form of microspheres using poly(lactic-*co*-glycolic acid) was approved by the FDA as the first atypical long-acting antipsychotic medication [22]. In the case of risperidone, (a second generation antipsychotic drug) [23,24] a combination of a low API dose with long-term treatment is needed in order to control the psychotic symptoms and treat schizophrenia. One major drawback of the marketed intramuscular (IM) injection depot formulation is the observed low initial drug release (<1% of dose) within the first three weeks of treatment. In order to overcome this, the simultaneous treatment of orally administrated risperidone, at least for this period of time, is recommended. In addition, repeated injections every two weeks are required in order to reach a steady-state API concentration within 6–8 weeks after treatment. The repeated injections may result in a higher rate of adverse reactions, such as the extrapyramidal symptoms (or else extrapyramidal side effects). Moreover, as the IM marketed formulation is not effective in the first three weeks, it cannot be considered as suitable for the treatment of short-term management (treatment for less than a month) [7,25]. Hence based on the above, the evaluation of new systems for the preparation of parenteral depot formulations that will effectively control the rate of API release, in order to overcome the aforementioned drawbacks in risperidone treatment, is of crucial importance.

In a previous study, poly(ε-caprolactone)/poly(propylene glutarate) (PCL/PPGlu) copolymer blends were evaluated as proper controlled release carriers for the preparation of risperidone transdermal patch formulations, with results showing that the synthesis and preparation of novel aliphatic polyester-based patches were able to optimize the drug dissolution profile [15]. Additionally, several other studies have attempted to overcome the marketed risperidone depot drawbacks, such as the development of a 3-month drug releasing risperidone formulation using polycaprolactones [26], or the reduction of drug’s release lag-period by either altering the microsphere’s preparation process [27,28], or by incorporating suitable bases (organic or inorganic) in PLGA based microspheres [29]. Therefore, it was the aim of the present study to evaluate, for the first time, the preparation of risperidone controlled release depot formulations based on novel polymer mixtures of PLA and poly(propylene adipate) (PPAd). For this reason, PPAd copolymers were synthesized by a two-stage melt polycondensation method and fully characterized. Microspheres of risperidone with neat PLA, neat PPAd, and blends of PLA/PPAd (prepared by a solvent evaporation method) at several weight ratios, were prepared using oil-in-water emulsification/solvent evaporation method and evaluated by scanning electron microscopy (SEM), Fourier-transform infrared spectroscopy (FT-IR), powder X-ray diffraction (XRD), and dissolution. In addition, an attempt to correlate the polyester’s enzymatic hydrolysis and API’s release rate with the aid of statistical moments was made for the first time.

## 2. Materials and Methods

### 2.1. Materials

Adipic acid (AdA, with purity above 99.5%), poly(phosphoric acid) (PPA), 1,3-propanediol (1,3-PDA) (purity: >99.6%), titanium butoxide (Ti(OBu)_4_, with purity of 97.0%) used as catalyst, and PLA (with mean *M*_n_ = 20,000 Da and polydispersity index (PDI) ≤ 1.3) were purchased from Sigma Aldrich Chemical Co. (Steinheim, Germany). Poly(vinyl alcohol) (PVA) with average *M*_w_ varying between 31,000 Da and 50,000 Da, 87%–89% hydrolyzed was also percussed from Sigma Aldrich Chemical Co. (Steinheim, Germany). Rhizopus delemar and Pseudomonas cepacia were purchased from BioChemika (Steinheim, Germany). Risperidone Drug, with particle sizes of D(0.10) = 10 μm, D(0.50) = 100 μm, and D(0.90) = 200 μm, was kindly donated by Pharmathen S.A. (Athens, Greece). All other reagents and solvents used for the analytical methods were of analytical grade.

### 2.2. Synthesis of Poly(Propylene Adipate) (PPAd) Aliphatic Polyesters

Two-stage melt polycondensation method (esterification and polycondensation) was used for the synthesis of PPAd aliphatic polyesters [30,31]. In brief, the correct amounts of adipic acid and 1,3-PDA (in a molar ratio 1/1.2) were charged into the reaction tube in the presence of Ti(OBu)_4_. The reaction mixture was heated at 180 °C in a nitrogen atmosphere, under continuous stirring, until the collection of almost all the theoretical amount of H_2_O. In the second step of polycondensation, PPA was added (5 × 10^−4^ mol of PPA/mol AdA) and vacuum (5.0 Pa) was applied slowly in order to reduce the creation of foam and the possibility of poly(adipic polyester) oligomer sublimation. Stirring was gradually increased and temperature of polycondensation remained stable at 240 °C for 2 h until the reaction was complete.

### 2.3. Poly(Lactic Acid)-Poly(Propylene Adipate) (PLA/PPAd) Blend Preparation

The solvent evaporation method was used for the preparation of PLA/PPAd blends. Specifically, appropriate amounts of PLA and PPAd (1 g whole amount), at different weight ratios were dissolved in 25 mL of dichloromethane and the solvent was allowed to evaporate under stirring at 25 °C. According to this procedure, several PLA/PPAd blends with weight ratios of 0/100, 20/80, 40/60, 60/40, 80/20, 100/0 *w*/*w* (PLA to PPAd) were prepared.

### 2.4. Characterization of Synthesized Polymers

#### 2.4.1. Intrinsic Viscosity

Intrinsic viscosity, η, measurement on the synthesized PPAd was performed by using an Ubbelohde viscometer at 25 °C. All polyesters were dissolved in chloroform (1% *w*/*v*) at room temperature and filtered through a 0.2 mm, Teflon filter and the intrinsic viscosity was calculated using Equation (1) [32]:

[η] = [2(*t*/*t*_o_ − ln(*t*/*t*_o_) − 1)]^1/2^/*c* (1)

where, *c* = concentration of the solution, *t* = flow time of solution, and *t*_o_ = flow time of pure solvent.

#### 2.4.2. Nuclear Magnetic Resonance (NMR)

1H NMR spectra of the synthesized PPAd was obtain in 5% *w*/*v* solutions with deuterated chloroform. Bruker spectrometer (Billerica, MA, USA) was used while the sweep width was 6 kHz, the operating frequency for protons was 400 MHz and a total of ten scans were collected.

#### 2.4.3. Gel permeation Chromatography

A Waters 150C gel permeation chromatography (GPC) system (Milford, MA, USA) equipped with differential refractometer was used for the analysis of synthesized PPAd. Three ultrastyragel columns (i.e., 103 Å, 104 Å, and 105 Å) were used in series, temperature was set at 35 °C, chloroform was selected as eluent, while the flow rate was set at 1 mL/min. Polystyrene standards of narrow *M*_w_ distribution were used for calibration.

#### 2.4.4. Wide Angle X-ray Scattering

X-ray powder diffraction (XRD) patterns of the neat PLA, PPAd, and the PLA/PPAd blends were recorded using an XRD-diffractometer (Rigaku, model MiniFlex 600, Chalgrove, Oxford, UK) with a CuKa radiation for crystalline phase identification. The samples were scanned from 5° to 60°.

#### 2.4.5. Differential Scanning Calorimetry (DSC)

DSC studies were performed in a Pyris Diamond DSC system (Perkin-Elmer, Dresden, Germany). Indium and Zinc standards were used for system calibration. Accurately weighed samples (5 ± 0.1 mg) of neat PLA, neat PPAd, and PLA/PPAd blends were sealed in aluminum pans and heated at 50 °C above their melting point at a heating rate of 20 °C/min. The samples were held at that temperature for 5 min in order to erase any thermal history and then were supercooled to −75 °C with a cooling rate of 200 °C/min. Subsequent heating scans of the quenched samples were recorded to observe the glass transition and melting temperatures.

#### 2.4.6. Enzymatic Hydrolysis

Polymer biodegradation describes the chain scission process during which polymer chains are cleaved in the form of oligomers and monomers resulting in polymer erosion and hence mass loss. In the present study, for the evaluation of the degradation process, polyesters (neat PLA, neat PPAd and PLA/PPAd blends) in the form of films (5 × 5 cm and ~2 mm thickness) were prepared by a PW 30 Otto Weber (Dusseldorf, Germany) hydraulic press connected to a temperature controller (Omron E5AX, Dusseldorf, Germany). The samples were incubated at 37 ± 1 °C for 25 days in suitable Petri dishes containing phosphate buffered saline (PBS) (pH = 7.4) with Rhizopus delemar and Pseudomonas cepacia lipases at 0.09 and 0.01 mg/mL content, respectively. After specific period intervals, the films were removed from the Petri dishes, washed with distilled water. and weighed until constant weight was achieved. The degree of biodegradation was estimated from the mass loss of the weighed polymer.

#### 2.4.7. Scanning Electron Microscopy (SEM)

SEM (JEOL JMS-840, Oxford Instruments, Tubney WoodsAbingdon, Oxfordshire, UK) was used to evaluate the morphology of the prepared films prior and after enzymatic hydrolysis. Carbon coating was used to cover the obtained films (improvement in the conductivity of the electron beam), while the accelerating voltage, the probe current, and the counting time were set at 20 kV, 45 nA, and 60 s, respectively.

### 2.5. Preparation of Risperidone Microspheres

Risperidone polymeric microspheres were prepared by the oil-in-water emulsification/solvent evaporation method. Briefly, 500 mg of the polyesters (neat PLA, neat PPAd or PLA/PPAd blends) were dissolved in 5 mL of dichloromethane (DCM), while 50 mg of risperidone (10% *w*/*w*) was dispersed under sonication for 1 min. The resultant oil-phase was homogenized with an aqueous phase (50 mL) consisting of 1% *w*/*v* poly(vinyl alcohol) (PVA), and then transferred to 100 mL of deionized water where it was left for 24 h at 25 °C under mild agitation. The resultant microspheres were collected after centrifuging at 8000× *g* rpm and washed three times with deionized water in order to remove the remaining quantities of DCM and PVA. Finally, the microspheres were freeze-dried in order to remove the excess of water and stored at 4 °C before further analysis.

### 2.6. Characterization Techniques

X-ray powder diffraction, differential scanning calorimetry, and scanning electron microscopy of the prepared microspheres were conducted similarly to the previously described analyses for the polymer blends (Section 2.4).

#### 2.6.1. Fourier-Transformed Infrared (FTIR) Spectroscopy

FTIR spectroscopy (FTIR-1000, Perkin Elmer, Dresden, Germany) was used to evaluate for possible interactions. All spectra (64 co-added scans) were collected from KBr-based discs, in the range of 450–4000 cm^−1^, at a resolution of 2 cm^−1^. The spectra presented were baseline corrected and converted to absorbance mode.

#### 2.6.2. Drug Loading, Yield, and Entrapment Efficiency (EE)

For the determination of drug loading, 1 mg of the prepared microspheres was dissolved in dichloromethane and the resultant solution was analyzed for assay by HPLC (method described in Section 2.6.5). Drug loading was calculated from Equation (2) as follows:

Drug loading (%) = (weight of drug in microspheres)/(weight of microspheres) × 100(2)

Microsphere yield was calculated by the following equation:

Yield (%) = (weight of microspheres)/(initial weight of polyesters and drug) × 100(3)

while, EE% was calculated by:

EE (%) = (weight of drug in microspheres)/(initial weight of drug) × 100(4)

#### 2.6.3. In-Vitro Drug Release

For the in-vitro release studies, a Distek Dissolution Apparatus (Evolution 2100C, North Brunswick Township, NJ, USA) was used equipped with an autosampler (Evolution 4300, North Brunswick Township, NJ, USA) using the basket method (United States Pharmacopeia, USP I method). Microspheres, placed into suitable dialysis tubing cellulose membranes (D9402-100FT, North Brunswick Township, NJ, USA) were inserted into the dissolution baskets, while the dissolution analysis was performed at 37 ± 1 °C with a rotation speed of 50 rpm. The dissolution medium was 500 mL of a phosphate buffered saline (PBS), pH = 7.4 solution, while sink conditions were not met in the present study. At predefined time intervals, 2 mL of aqueous solution was withdrawn from the release media and analyzed for risperidone content by HPLC (the method is described below).

#### 2.6.4. Statistical Moments

Statistical moments were used as a correlation approach for hydrolysis and dissolution release processes. Statistical moments, used as curve-fitting algorithms [33] are defined as the expected value of a positive integral power of a random variable. In the present study correlation of the polyester enzymatic hydrolysis and Active Pharmaceutical Ingredient (API) dissolution processes were evaluated by the first statistical moment about zero:

(5) $$M\left(X\right)T=\frac{\int t\left(X\left(t\right)\right)dt}{\int X\left(t\right)dt}$$

where, *X*(*t*) is the time-dependent variation of the examined process, and *M*(*X*)*T* is the mean process time. In the case of hydrolysis, *M*(*X*)*T* represents the time at which the 63.2% of polyester mass was lost; while in the case of dissolution it represents the time at which the 63.2% of the drug was released from the matrix.

#### 2.6.5. HPLC Method

Risperidone content was assayed using a Shimadzu Prominence HPLC system consisting of a degasser (Model DGU-20A5, Tokyo, Japan), a pump (Model LC-20AD, Tokyo, Japan), an autosampler (Model SIL-20AC, Tokyo, Japan), a UV-Vis detector (Model SPD-20A, Tokyo, Japan), and a column oven (Model CTO-20AC, Tokyo, Japan). Chromatographic analysis was performed with a C_18_ column (CNW Technologies Athena, 120 Å, 5 μm, 250 mm × 4.6 mm, Tokyo, Japan) at 25 °C. Methanol/H_2_O/triethylamine at a ratio of 80/19.5/0.5 *v*/*v*/*v* was used as mobile phase, while acetic acid was used to adjust the pH at 10.22. The flow rate was set at 1.0 mL/min, injection volume was 20 μL, while the risperidone analysis was performed at 254 nm.

## 3. Results and Discussion

### 3.1. Characterization of Prepared Polyesters

#### 3.1.1. Synthesis and Characterization of PPAd

**Figure 1:**
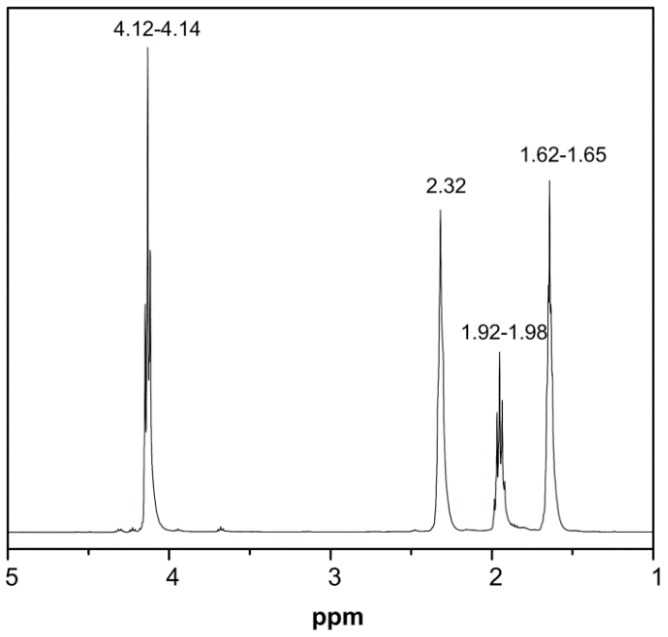
Hydrogen-1 nuclear magnetic resonance (1H-NMR) spectra of the newly synthesized poly(propylene adipate) polyester.

The synthesis and characterization of the PPAd polyesters from the reaction of 1,3-PDA in the presence of Ti(OBu)_4_ (working as a catalyst) were analytically discussed in a previous work of ours [34]. Briefly, in the first stage oligomers of PPAd were synthesized by removing water as byproduct, whereas in the second stage the molecular weight of the polymer was augmented by increasing the reaction temperature, while water and 1,3-PDA byproducts were removed. Characterization of the newly synthesized PPAd polyester by gel permeation chromatography and intrinsic viscosity measurements showed a high molecular weight value (*M*_n_ = 13,700 Da) and an intrinsic viscosity of 0.57 dL/g. In a further step, 1H NMR spectra for PPAd in Figure 1 showed several peaks which were in agreement with previous published data [34]. Specifically, three peaks corresponding to the methylene group e were recorded in the region of 4.12–4.14 ppm, five peaks corresponding to the methylene group f were recorded in the region of 1.92 to 1.98 ppm, a single peak corresponding to the methylene group g was recorded at 2.32, while another five peaks corresponding to the methylene group h were recorded at 1.62–1.65.

#### 3.1.2. Thermal Analysis

**Figure 2:**
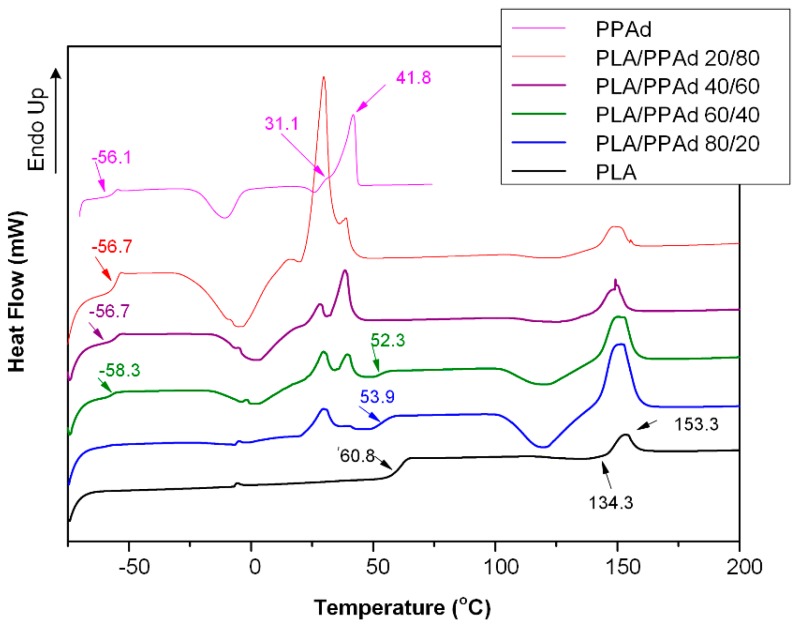
Differential scanning calorimetry (DSC) thermograms of pure poly(propylene adipate) (PPAd), poly(lactic acid) (PLA) and poly(lactic acid)-poly(propylene adipate) (PLA/PPAd) blends at various weight ratios.

Aliphatic polyesters, as well as their copolymers or blends, are widely used as drug carriers in the preparation of depot drug formulation due to their cytotoxicity and biocompatibility. Generally, polyester blends exhibit various thermal behaviors which depend on their miscibility or compatibility. Figure 2 shows the DSC thermograms of the newly synthesized PPAd polyester, the neat PLA, and the PLA/PPAd blends after quenching from their melts. Regarding PPAd, the DSC thermogram showed a low glass transition temperature (*T*_g_) at −56.1 °C, while during heating, the polyester begins to crystallize with a cold crystallization temperature (*T*_cc_) at −8 °C. Almost immediately after crystallization, melting of the polymer starts with two endothermic overlapping peaks recorded at 31.1 °C (pre-melting) and 41.8 °C (main melting), respectively. In the case of neat PLA, a *T*_g_ value was recorded at 60.8 °C, a cold crystallization at 134.3 °C, while a pure melting endotherm was also recorded at 153.3 °C. With regard to the polyester blends, all mixtures showed two melting peaks ascribed to the neat polymers, indicating the prepared blends can be classified as (semi)crystalline/(semi)crystalline immiscible systems. Some differences recorded in the *T*_g_ values of the PLA/PPAd blends compared to the neat PLA, were due to the in-situ melting of PPAd which results in a lowering of the PLA’s *T*_g_ value.

#### 3.1.3. X-ray Diffraction Studies

**Figure 3:**
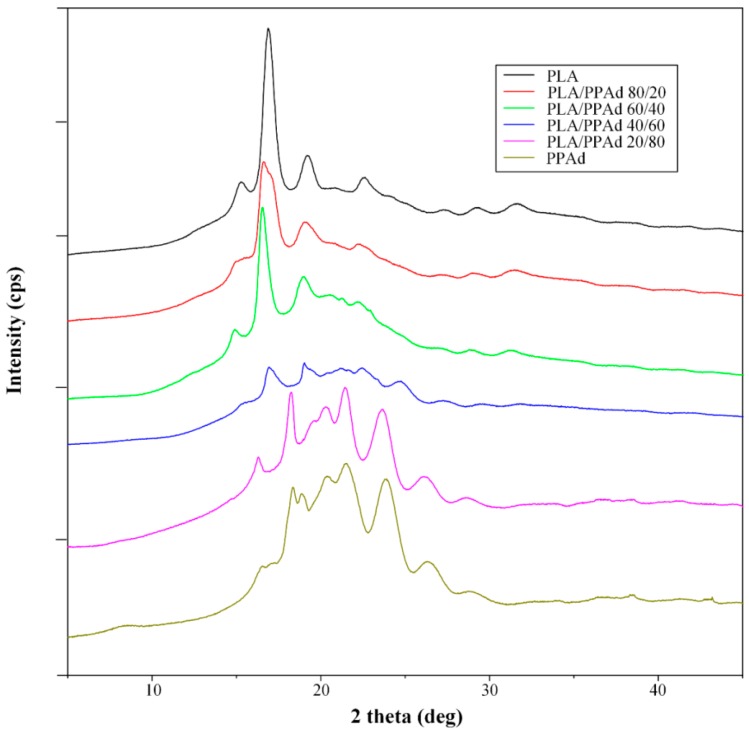
X-ray diffraction (XRD) pattern of the pure PLA and PPAd along with the prepared PLA/PPAd polymer blends at different weight ratios.

The above DSC studies indicated that PLA/PPAd blends are immiscible, as distinct melting peaks corresponding to each polymer were recorded. This was also confirmed from the XRD studies shown in Figure 3. Generally, in (semi)crystalline/(semi)crystalline polymer blends the peak intensities recorded in the XRD patterns depend on the concentration of each polymer, as both components crystallize separately. In the present study, the X-ray diffraction pattern of neat PLA showed two characteristic main peaks at 2θ 16.8° and 19.5°, while neat PPAd showed several characteristic peaks at 2θ 18.70°, 20.76°, 21.92°, 24.25°, and 26.93°. Comparison of the XRD patterns for both neat polymers showed increased crystallinity for PLA, compared to PPAd, indicating that the two-stage melt polycondensation method used for the synthesis of PPAd leads to the formation of a partially amorphous polyester. When examining the XRD patterns of PLA/PPAd polymeric blends, the corresponding diffraction peaks of both neat polyesters were recorded in all cases, while the peak intensities depended on the concentration of each polymer; indicating that the two polyesters were immiscible and crystallize separately within the polymeric blend.

#### 3.1.4. Hydrolysis Rate

**Figure 4:**
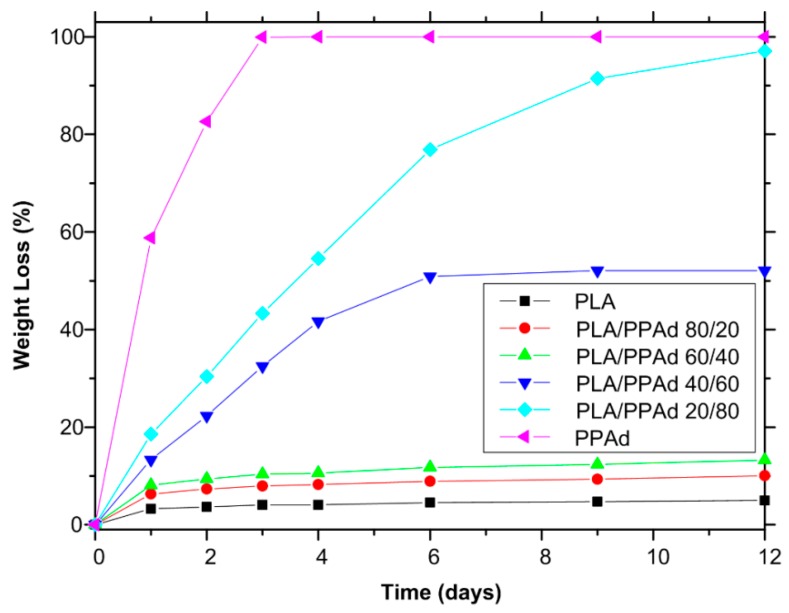
Weight loss versus time of neat PLA, neat PPAd and PLA/PPAd blends at various weight ratios during hydrolysis test in the presence of enzymes at 37 °C and pH 7.4.

Polyester erosion through cleavage via hydrolysis of the ester groups is one of the main mechanisms of drug release from such polymeric-based systems. Hence, the evaluation of hydrolysis is of crucial importance. Keeping in mind, however, that the in vivo hydrolysis of PLA is very slow and hence time-consuming [35], it is a common practice to evaluate the process in the presence of enzymes [36]. Specifically, the enzymatic hydrolysis of polyester was studied using a mixture of *Rhizopus delemar* and *Pseudomonas cepacia* lipases. Lipases are able to cleave ester bonds in the solid phase since they can be activated by adsorption on hydrophobic surfaces. As can be seen from Figure 4, neat PLA showed only limited mass loss during hydrolysis in the presence of enzymes, with approximately 2.0% of the initial mass being lost within the first two days. No further change in PLA mass loss was recorded for up to 12 days. On the contrary, PPAd was fully hydrolyzed within only three days in the presence of enzymes, indicating that the prepared polyester is fully biodegradable in short time. The different behavior of polyesters during hydrolysis can be ascribed to several factors such as differences in the molecular weight, hydrophilic/hydrophobic balance segments within the main chain, degree of crystallinity and crystal morphology etc. [37,38,39]. With regard to PLA/PPAd blends, the recorded mass loss rates during enzymatic hydrolysis were in between the rates recorded for the neat polyesters. Specifically, results in Figure 4 showed that as PPAd content increases in the polyester blend, the enzymatic hydrolysis rate increases, with approximately 80%, 50%, and 10% of mass loss being recorded within six days for blends having 80%, 60%, and 40% *w*/*w* PPAd, respectively. Hence, based on the above results, it can be concluded that polymer erosion through enzymatic hydrolysis may be manipulated by adjusting the content of PPAd in the mixture.

**Figure 5:**
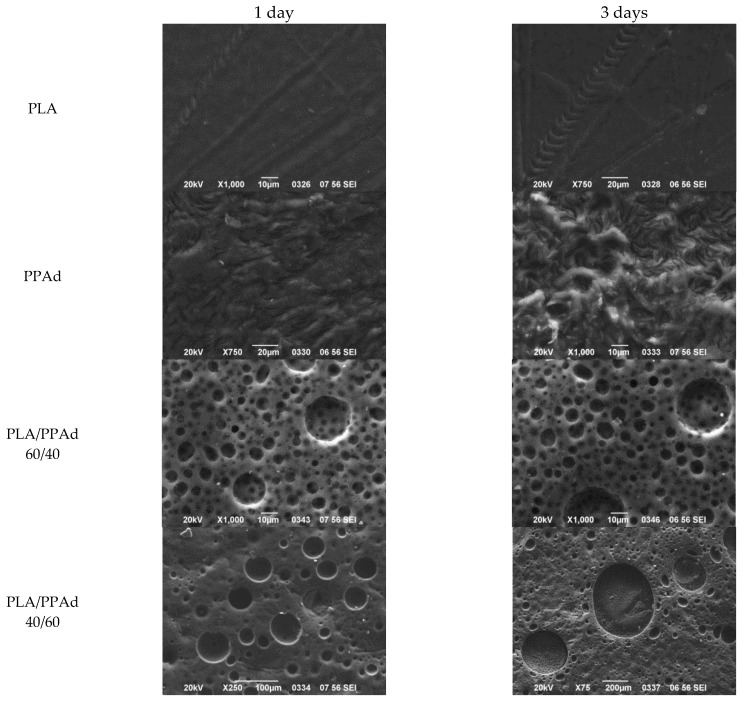
Scanning electron microscopy (SEM) pictures for surfaces after one and three days of enzymatic hydrolysis: neat PLA, neat PPAd, PLA/PPAd 60/40 and PLA/PPAd 40/60 *w*/*w*.

Although the analysis of the weight loss profiles given in Figure 4 depicts a general trend for the rate of enzymatic hydrolysis, it does not, however, show how this hydrolysis proceeds within the polymer matrices. Hence, in order to gain a better insight into the enzymatic hydrolysis process morphological examination of the samples was performed. Figure 5 shows the specific features of the enzymatically hydrolyzed surfaces of polyester specimens as recorded by SEM micrographs at one and three days of hydrolysis.

The SEM micrograph of neat PLA after one and three days of hydrolysis revealed a homogeneous and rather smooth surface, indicating good hydrolytic stability for the polymer. This can be attributed to the increased hydrophobic nature of the polyester, compared to the PPAd, where extended degradation was observed already from the first day of examination. With regard to PLA/PPAd blends, structural deterioration on the surface texture of the films was observed from the first day of the enzymatic hydrolysis test on the entire surface. Comparison of the blends with different content of PPAd showed that as the PPAd increased, the size of the pores created by the hydrolysis process also increased. Specifically, at the third day of hydrolysis films having 40% *w*/*w* of PPAd showed pores with diameter up to 20 μm, while films having 60% *w*/*w* of PPAd showed pores with diameter up to 400 μm at the same time interval. These pores belong mainly to hydrolyzed PPAd, which, due to its immiscibility with PLA, creates separated phases inside the polymeric matrix.

### 3.2. Characterization of Risperidone Drug Formulations

#### 3.2.1. Microsphere Morphology

**Figure 6:**
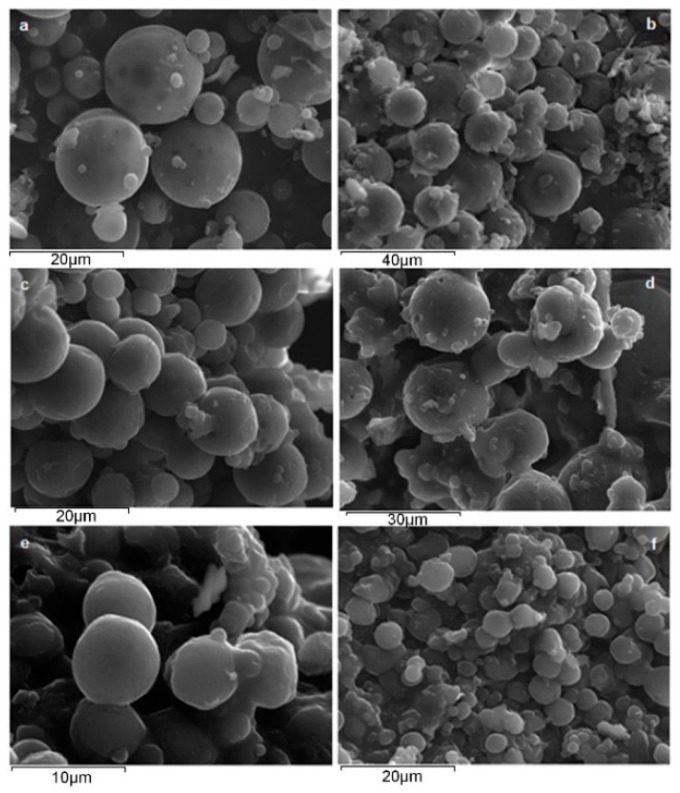
SEM pictures of risperidone microspheres prepared by neat PLA (**a**), PLA/PPAd 80/20 *w*/*w* (**b**), PLA/PPAd 60/40 *w*/*w* (**c**), PLA/PPAd 40/60 *w*/*w* (**d**), PLA/PPAd 20/80 *w*/*w* (**e**) and neat PPAd (**f**).

Figure 6 shows the morphology of the prepared risperidone microparticles obtained by SEM. In the case of neat PLA the prepared microparticles showed a spherical morphology with sizes varying from 2.5 to 15 μm, while neat PPAd microparticles were smaller in size (varying from 1 to 5 μm) with less polydispersity. Additionally, in the case of PPAd microparticles less spherical particles and an increasing number of agglomerates were observed compared to neat PLA particles. This is due to the softness of PPAd and to its low glass transition temperature. No significant differences were observed between the risperidone microparticles prepared by different weight ratios of PLA to PPAd, while all prepared microparticles had a smooth surface with no cracks or pores. Their size ranged between 2 and 17 μm without big differences among the different polymer ratios. Some smaller particles were prepared only for PPAd neat polyester.

#### 3.2.2. API Physical State Characterization

**Figure 7:**
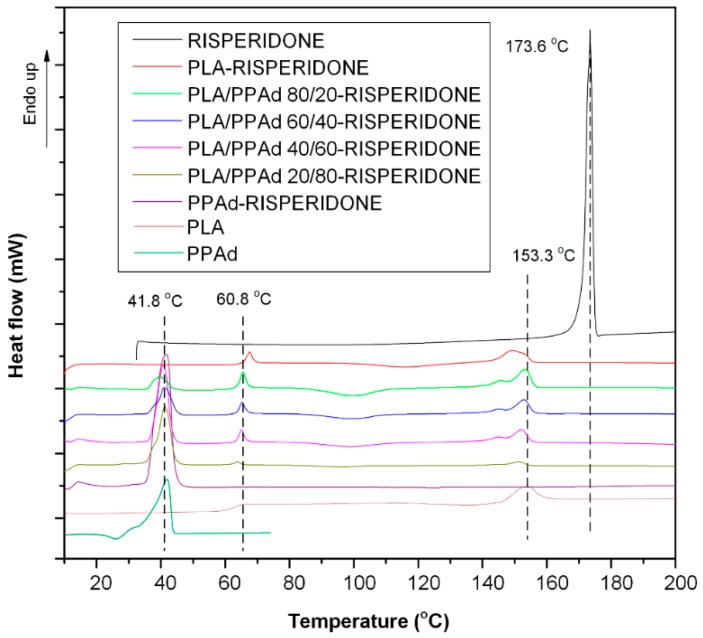
DSC thermograms of neat risperidone, PLA and PPAd, along with the prepared risperidone microspheres with PLA, PPAd, and PLA/PPAd blends at several weight ratios.

**Figure 8:**
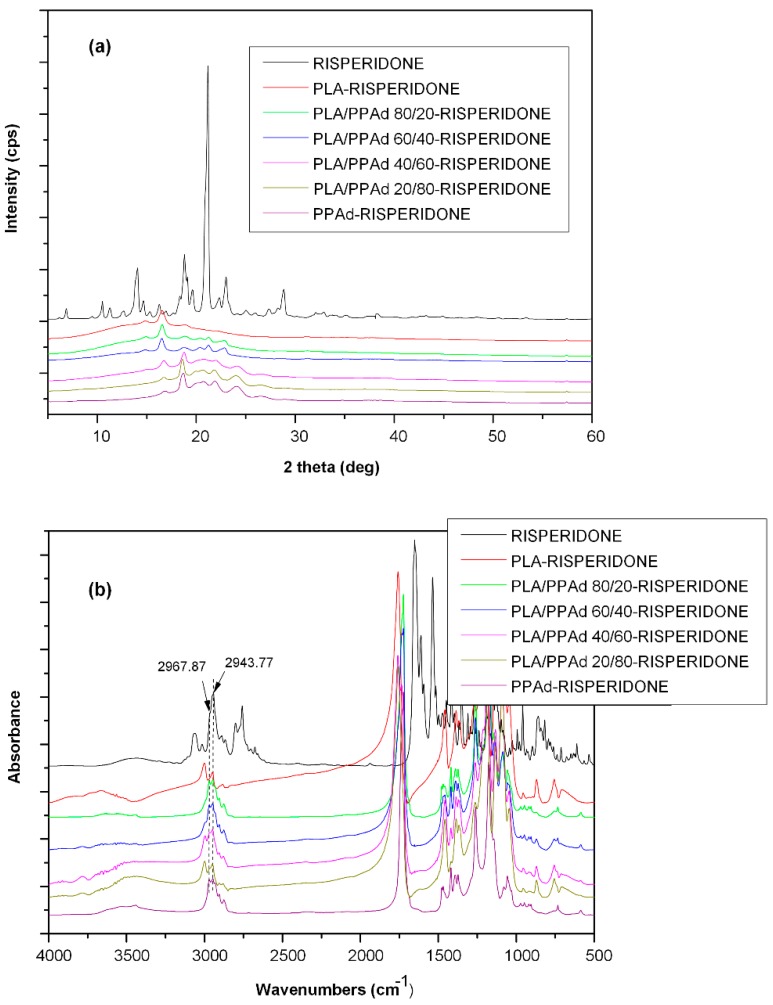
X-ray diffraction (XRD) patterns (**a**) and Fourier-transformed infrared (FTIR) spectra (**b**) of neat risperidone, risperidone–PLA and risperidone–PPAd microspheres along with risperidone microspheres with PLA/PPAd blends at various weight ratios.

As drug dissolution is controlled by both polymer erosion and drug diffusion, evaluation of API’s physical state within the prepared microspheres plays an important role. In addition, API solid state properties and physical state have an important role in the chemical and physical stability of the prepared formulations. Hence, the physical state of the API within the prepared polyester microspheres was evaluated by several techniques. Initially, the thermal properties of the prepared systems were evaluated via DSC. Specifically, Figure 7 shows the DSC thermograms of neat risperidone, PLA, and PPAd along with the prepared risperidone/polyester-based microspheres. From the results, it is clear that the thermal behavior of microspheres is similar to that of neat polyesters and their blends (Figure 2) with some small differences. In all cases the characteristic melting points and glass transition temperatures of the neat polyester were recorded, indicating that the prepared microspheres consisted of immiscible PLA/PPAd blends, while the effect of API on polymer blend thermal properties is limited, as no significant changes compared to neat polyester blends were observed. Furthermore, as can be seen from the thermographs, the risperidone sharp endothermic peak at 173.6 °C (corresponding to its melting point) was not recorded in the prepared microsphere formulations, in which only the melting characteristics of the two polyesters (PLA and PPAd) were seen. This is an indication that the drug is probably dispersed in the amorphous phase within the polymer matrices. It is important, however, to note that based on the given thermograms it is not possible to exclude the in-situ amorphization of API within the DSC aluminum pan due to the solubilization (or partial solubilization) of the drug crystals into the neat melted polyesters or the melted polyester blends. Hence, in order to verify the amorphous state of the API, all samples were studied by XRD and the recorded patterns are presented in Figure 8a.

From the XRD analysis, although pure risperidone showed significant crystallinity, the patterns of all prepared microspheres showed only the characteristic peaks of the two polyesters, verifying the DSC’s findings, that the API is dispersed within the polymers at a molecular level, while a significant reduction on the degree of polyester crystallinity was also observed.

In a further step, and in order to evaluate whether there are any interactions taking place between the API and the tested polyesters, FTIR spectroscopy was used (Figure 8b). Regarding risperidone’s spectra, a weak band at 3064 cm^−1^ corresponding to the C–H stretching of the aromatic ring was recorded, while two peaks at 1643 cm^−1^ and 1612 cm^−1^ were shown, indicative of the C=O stretching of the δ-lactam ring and the C=C stretching, respectively [40]. FTIR spectra of risperidone-loaded microspheres with either neat polyesters or PLA/PPAd blends were the sum of the individual component spectra, indicating that there are no interactions taking place among the API and the examined polyesters. Hence, it can be assumed that the API amorphization observed by DSC and XRD analysis is probably due to homogeneous dispersion of risperidone within the polymer matrix blends rather than the formation of H-bonds between functional groups of the studied components.

#### 3.2.3. Drug Loading, Yield and % EE

Microsphere yield, drug loading content and % EE are presented in Table 1. Yield varied from 78.34% to 82.37% indicating high process efficacy, while drug loading varied from 9.84% (in the case of pure PLA) to 14.21% (in the case of PLA/PPAd 40/60) and % EE varied from 42.82% (in the case of pure PPAd) to 36.51% (in the case of PLA). Generally, several factors may affect both drug loading and % EE, such as the affinity of the loaded drug with the polyesters, the hydrophobicity of the polymer matrix, drug solubility in water, drug-drug interaction (i.e., its ability to self-aggregate) etc. [30]. Specifically, in the present study, % EE and drug loading seem to increase with increasing PPAd content, indicating that the amorphous and more hydrophilic nature of the specific polyester enhances process efficacy compared to PLA.

#### 3.2.4. Dissolution Studies Results

**Figure 9:**
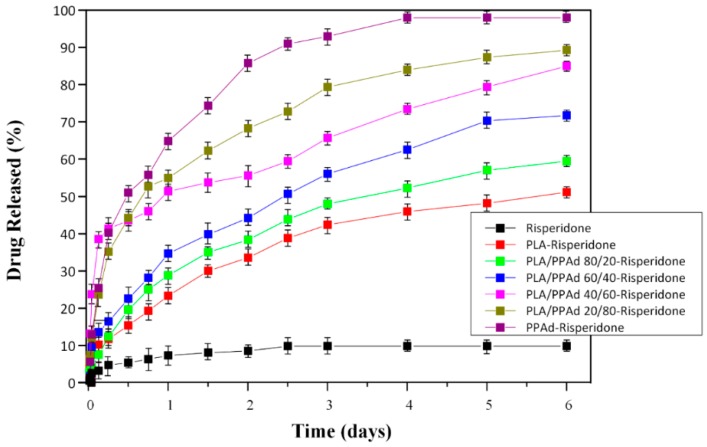
In vitro release profiles of neat risperidone, along with risperidone-loaded microspheres prepared from PLA, PPAd and PLA/PPAd blends.

With respect to API-microsphere dissolution profiles, results in Figure 9 showed a very low dissolution rate when pure API was tested alone (not exceeding 10% risperidone release even after six days of testing), probably due to the high API hydrophobicity. In contrary, all microsphere formulations showed much higher dissolution release rates, which can be attributed to the amorphous or molecular level dispersion of risperidone inside the polymer matrix. In all cases, risperidone release from prepared microspheres followed two-step release kinetics, i.e., an initial burst release phase, observed up to approximately six hours (indicating that some portion of the API is probably bonded on the surface of the microspheres), and a controlled release phase which lasted till the end of dissolution process. In addition, dissolution results showed that microspheres consisting of neat PPAd release up to 95% of the API within the first three days of dissolution, while at the same time only the 40% of the API is released by neat PLA microspheres. These differences may be attributed to several factors, including water permeability and solubility within the polymer matrix, degree of crystallinity and crystalline morphology of polymers (since the API is in amorphous state in both cases), hydrophilic/hydrophobic balance of the two polyesters, particle size and size distribution of the prepared microspheres, drug loading levels etc. With regard to dissolution release profiles of API-loaded microspheres prepared by PLA/PPAd blends, dissolution release rates in-between the two “extremes” (i.e., neat PLA and PPAd) were observed as expected. By increasing the PPAd amount in the PLA/PPAd blends the API release rate also increased. This was probably due to the low melting point and low glass transition temperatures of PPAd compared to PLA, verified also by a previous work of ours, where both (*T*_m_ and *T*_g_) were identified as having an important role in drug release rate [30].

Before moving ahead with the analysis of the drug release mechanism it is important to evaluate the above results in terms of optimum composition. As stated in the introduction one of the aims of the present study was to evaluate PLA/PPAd blends as carriers in the preparation of risperidone long-acting injectable formulations suitable for short-term treatment. Based on the analysis of dissolution profiles (Figure 9) the preparation of risperidone formulations with the use of neat PLA and PPAd did not result in the desirable outcome. Specifically, microspheres based on pure PPAd showed a relatively fast dissolution profile with approximately 85% of the API being released in two days, while in the case of pure PLA, risperidone microspheres showed a relatively slow dissolution rate with less than 50% of the API being released within the first six days. On the contrary, the use of PLA/PPAd blends showed dissolution profiles in-between the two extremes, which makes them ideal candidates for preparing depot formulations suitable for risperidone short-term treatment. Specifically, by changing the ratio of PLA to PPAd a wide variety of dissolution profiles was obtained, indicating that the proposed PLA/PPAd based formulations may provide the basis for the preparation of a novel pharmaceutical product platform which could cover risperidone’s short-term treatment from a few days (PLA/PPAd ratio of 20/80) up to a few weeks (PLA/PPAd ratio of 80/20).

#### 3.2.5. Statistical Moment Analysis

In a further step, in an attempt to correlate the drug release rate with the polymer hydrolysis rate statistical moments were used as curve-fitting algorithms. For this reason, the first statistical moment about zero of the polyester mass loss, and the API dissolution were calculated (Table 2). Results showed that both statistical moment values increase as the content of PLA increases, while in the case of hydrolysis the values reach a plateau at approximately 300 h.

**Figure 10:**
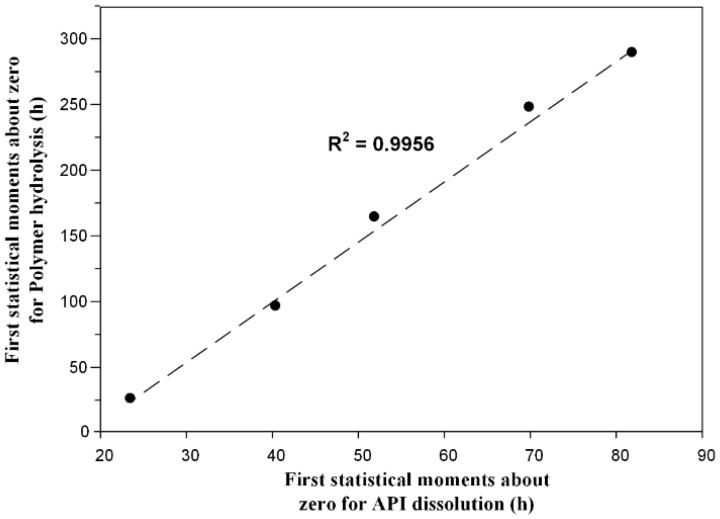
Correlation diagram of the first statistical moments about zero for the polyester hydrolysis and active pharmaceutical ingredient (API) dissolution processes.

Figure 10, shows the correlation scatter plot between the first statistical moments about zero for the polyester hydrolysis and API dissolution processes, where a linear correlation between the two moments, with an *R*^2^ value of 0.9956 was observed, indicating a one-to-one correlation between the polymer enzymatic hydrolytic degradation and the API dissolution processes.

#### 3.2.6. Release Data Modeling

Initially, an attempt was made to characterize the release kinetics of the pure API, which in general, consists of two distinct steps. The first step is an apparent reaction step, during which the solid API is transformed to its dissolved form, while the second step (highly controlled by stirring intensity) is an external mass transfer step where the dissolved API is transferred into the bulk liquid. In fact, for similar API dissolution trials using the same stirring conditions as the ones selected in the present study, it can be argued that there is no significant contribution of the external mass transfer step and hence the API dissolution process is controlled by the apparent reaction step (e.g., aprepitant [41], paliperidone [42], and paclitaxel [43]). However, this is not the case for risperidone, where initially the API release curve increases very quickly and then continues with a rate which is slower than the one corresponding to first-order kinetics. In this case, it is more appropriate to consider the pure API dissolution process as having a general *n*-th order kinetics (Equation (6)).

(6) $$\frac{dX}{dt}=-K{\left(1-X\right)}^{n}$$

where, *X* denotes the ratio between actual drug concentration (*C*) and solubility (*C*_eq_) and *K* is the kinetic constant. After integration using *X*(0) = 0 the above equation leads to Equation (7):

(7) $$X=1-{\left(1+\left(n-1\right)kt\right)}^{1/\left(1-n\right)}$$

**Figure 11:**
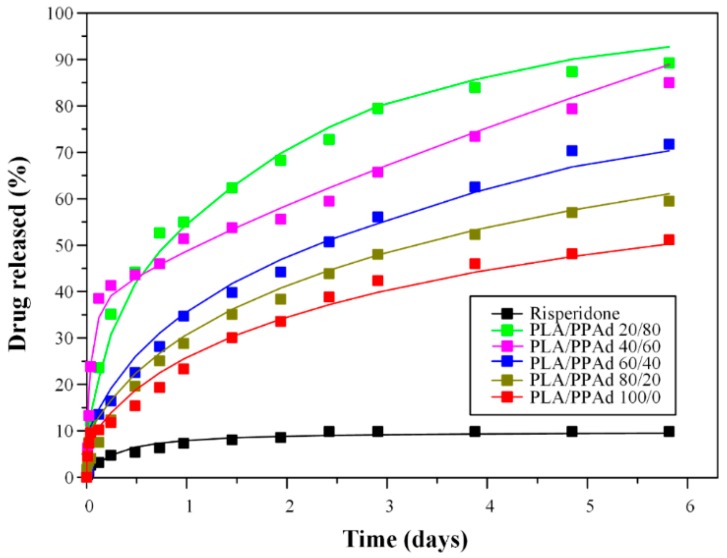
Comparison between experimental and model drug release evolution. The experiments are shown as data points and the models as continuous lines.

The above *X* evolution curves can be transformed into the release profile curves shown in Figure 9 by multiplying with *X_f_* = (*C*_eq_ × *V*)/*m* (where, *V* is the liquid volume and *m* is the total drug mass). Fitting of the pure API dissolution results on the above *n*-th order dissolution release model (Figure 11 with a black line) showed much better results compared to the first-order fitting, while the calculated fitting parameters were *X_f_* = 0.098, *K* = 4 × 10^−5^ s^−1^, and *n* = 1.86.

**Figure 12:**
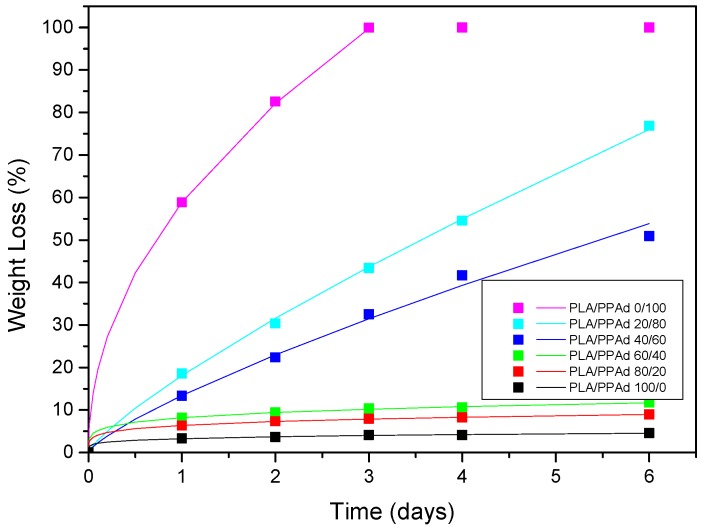
Weight loss evolution of the polymer composites during hydrolysis. Experimental data are shown with squares and power-law fits with lines.

In a further step, API dissolution modelling was attempted for the risperidone-loaded microsphere formulations. In order to be more concise with the description of the results, the microspheres prepared from PLA/PPAd blends with analogies 0/100, 20/80, 40/60, 60/40, 80/20, and 100/0 are denoted in the following analysis as formulations I, II, III, IV, V and VI, respectively. Based on the previous statistical moment analysis a one-to-one correlation between polymer erosion and API dissolution process was revealed, indicating that the polymer erosion process is of crucial importance. Hence, in order to proceed with the drug release modelling a quantitative description of the erosion kinetics was attempted. In general, direct modelling of the erosion process is not possible as it is a very complex process governed by several factors such as water diffusion into the matrix, matrix swelling, reaction between water and polymer, diffusion of oligomers and monomers produced, geometric considerations (volume or surface reaction) etc. [44]. In the present study modelling of the erosion process was attempted using the data from zero up to six days, since the API dissolution experiments were conducted for the same time interval. Fitting of the obtained results in Figure 12 showed that the use of a simple power law, such as *WL* = *A* × *t^n^* (where *WL* is the polymer weight loss during hydrolysis, and *A* and *n* are fitting parameter), describes well all experimental data. The fitting parameters were: *A* = 58.9, 18.13, 13.43, 8.18, 6.38, and 3.26, and *n* = 0.48, 0.8, 0.775, 0.2, 0.19, and 0.18 for blends I, II, III, IV, V, and VI, respectively.

However, before proceeding to the API dissolution modelling a close inspection/comparison of Figure 4 and Figure 9 (hydrolysis and dissolution profiles, respectively) shows that the API release fraction is larger compared to the corresponding polyester erosion fraction, suggesting that polymer erosion is not the only release mechanism governing risperidone’s dissolution process. In such cases, drug from the non-eroded part of the polymeric matrix diffuses in the liquid following either a Fickian or a non-Fickian diffusion process [45]. Since, the scope of the present analysis is to develop the simplest possible models that can adequately describe the data and in parallel are compatible with the underlying physical mechanisms, the studied microsphere formulations were divided into those controlled by: (1) mainly erosion (formulation I), (2) mainly diffusion (formulations IV, V, and VI), and (3) an equal contribution of erosion and diffusion processes (formulations II and III). 

In the case of risperidone-loaded microsphere formulations consisting of neat PPAd (formulation I) the experimental curves of hydrolysis and drug release are comparable with each other, and hence, the release mechanism can be considered as being controlled only by polyester erosion. On the other hand, for formulations IV, V, and VI, it seems that only a small portion of API is released due to polymer erosion (approximately 8% to 18%) and hence, in order to keep the model simple, diffusion was considered as the only release mechanism for these trials. In these cases (i.e., formulations IV, V, and VI) a first-order release model, corresponding to an approximation of the Fickian-diffusion process, was initially tested [46], however the essential inability of this simple model to fit adequately the experimental data corresponding to the initial API burst release led to the following modification [47]:

(8) $$X=X_{f}\left(1-\frac{6}{\pi^{2}}\overset{\infty }{\underset{i=1}{\sum }}\frac{1}{i^{2}}\exp\left(-ki^{2}\pi^{2}t\right)\right)$$

where, *X* is the fraction of the drug that has been released at time t, and k is a release fitting parameter that has inverse time units and can be computed as *k* = *D*/*R*^2^ (where *D* is the Fickian diffusivity of the drug into the polymer, *R* is the radius of the prepared microspheres), and *i* is the counter of the sum which takes values from one to infinity. *X* in Equation (9) is transformed to percentage of drug released by multiplying by 100.

In contrast to the above cases, formulations II and III could not be adequately described by assuming a sole release mechanism; and hence, in these cases, both diffusion and erosion processes should be considered during dissolution release modelling. Specifically, Figure 9 shows that the release profile of formulation III does not follow the trend of the rest formulations, as an extremely fast API burst effect step observed in the first time points is followed by a constant dissolution rate. Hence, in the case of formulation III the modification of the above diffusion model (Equation (8)), by adding the contribution of erosion, seems to be more appropriate (Equation (9)):

(9) $$X=X_{f}\left(1-\frac{6}{\pi^{2}}\overset{\infty }{\underset{i=1}{\sum }}\frac{1}{i^{2}}\exp\left(-ki^{2}\pi^{2}t\right)\right)+WL/100$$

In contrary to the above case, where the diffusion and erosion processes are considered to act independently, formulation’s II release profile seems to be controlled simultaneously by both the diffusion and erosion processes. In this case, erosion affects diffusion by reducing the particle size of the microspheres while diffusion affects erosion by reducing the concentration of the drug released as the erosion process proceeds. An approximate model that takes into account all the above phenomena is given in the following equation:

(10) $$X=1-\left(1-\frac{WL}{100}\right)\left[\left(\frac{6}{\pi^{2}}\overset{\infty }{\underset{i=1}{\sum }}\frac{1}{i^{2}}\exp(-k{\left(1-\frac{WL}{100}\right)}^{-\frac{2}{3}}i^{2}\pi^{2}t)\right)X_{f}+\left(1-X_{f}\right)\right]$$

Hence, the release curves for formulations IV, V, VI were fitted using Equation (8), for formulation III using Equation (9) and for formulation II using Equation (10), by employing a least square deviation minimization procedure. The comparison between experimental data and model curves is presented in Figure 11. The fitting performance can be considered acceptable (*R*^2^ > 0.95), taking into account the uncertainty of the experimental data. The only question that remains unanswered is why formulation III shows a different dissolution behavior compared to the rest of the PLA/PPAd blend formulations, since its composition is slightly different from the rest. A possible explanation (without invoking a different release mechanism) is probably the non-uniform distribution of the API within the formed polymeric microspheres (occurring for unknown reasons only in this case), as it is well known that the API release behavior is very sensitive to drug distribution [48]. The fitting parameters for blends II, III, IV, V, VI were *X_f_* = 0.88, 0.35, 0.84, 0.81, 0.63 and *k* = 3.2 × 10^−6^ s^−1^, *k* = 1.74 × 10^−4^ s^−1^, 2.31 × 10^−6^ s^−1^, 1.82 × 10^−6^ s^−1^, *k* = 2.1 × 10^−6^ s^−1^, respectively. Additionally, it is important to note that the above analysis revealed a clear pattern of increasing *X_f_* and decreasing *k* values as PLA content increased (with the exception of formulation III).

## 4. Conclusions

In the present study, risperidone microspheres with various PLA/PPAd blends were successfully prepared. Characterization of the prepared blends showed that the two polyesters were immiscible at all weight ratios examined, while PPAd showed remarkably increased enzymatic hydrolytic degradation compared to PLA. Risperidone was dispersed in the amorphous state within the polymer matrix, while in-vitro release studies showed that the API release rate varied according to the weight ratio, and hence the properties of the used polymer blends. Application of statistical moments showed the polyester degradation process (erosion) to be an important mechanism of API release, while API release kinetics modeling showed diffusion as the main mechanism of release in polymer blends having a high PLA content. PLA/PPAd blends could be a novel pharmaceutical platform which could cover risperidone’s short-term treatment from a few days (PLA/PPAd ratio of 20/80) up to a few weeks (PLA/PPAd ratio of 80/20).

## Figures and Tables

**Table 1:** Particle size, yield, drug loading content and % entrapment efficiency (EE) of risperidone PLA/PPAd prepared microspheres.

Sample	Particle Size Range (μm)	Microparticles Yield (%)	Drug Loading (%)	Entrapment Efficiency (%)
PLA–risperidone	3–17	78.34 ± 2.1	9.84 ± 1.7	36.51 ± 2.2
PLA/PPAd 80/20-risperidone	3–15	79.56 ± 2.1	11.24 ± 3.1	38.46 ± 3.1
PLA/PPAd 60/40-risperidone	3–14	82.37 ± 1.9	12.87 ± 3.0	41.87 ± 3.2
PLA/PPAd 40/60-risperidone	3–15	81.48 ± 2.9	14.21 ± 2.5	40.42 ± 2.6
PLA/PPAd 20/80-risperidone	2–10	80.54 ± 3.0	10.07 ± 3.1	39.17 ± 4.1
PPAd-risperidone	2–8	82.17 ± 2.0	12.49 ± 1.9	42.82 ± 3.0

**Table 2:** Statistical moment parameters for polyester enzymatic hydrolysis (mean weight loss time) and risperidone dissolution (mean active pharmaceutical ingredient (API) dissolution time).

Polyester Type	First Statistical Moment about Zero for:	
	Dissolution (h)	Hydrolysis (h)
PLA	90.25	300.72
PLA/PPAd 80/20	81.85	300.24
PLA/PPAd 60/40	69.90	256.80
PLA/PPAd 40/60	51.89	169.92
PLA/PPAd 20/80	40.39	99.36
PPAd	23.50	26.16

## References

[1] Baweja R., Sedky K., Lippmann S. (2012). Long-acting antipsychotic medications. Curr. Drug Targets.

[2] Schwendeman S.P., Shah R.B., Bailey B.A., Schwendeman A.S. (2014). Injectable controlled release depots for large molecules. J. Control. Release.

[3] Wan F., Yang M. (2016). Design of PLGA-based depot delivery systems for biopharmaceuticals prepared by spray drying. Int. Pharm..

[4] Fleischhacker W.W. (2009). Second-generation antipsychotic long-acting injections: Systematic review. Br. J. Psychiatry.

[5] Chaurasia S., Mounika K., Bakshi V., Prasad V. (2017). 3-month parenteral PLGA microsphere formulations of risperidone: Fabrication, characterization and neuropharmacological assessments. Mater. Sci. Eng. C Mater. Biol. Appl..

[6] Remenar J.F. (2014). Making the Leap from Daily Oral Dosing to Long-Acting Injectables: Lessons from the Antipsychotics. Mol. Pharm..

[7] Tian W., Schulze S., Brandl M., Winter G. (2010). Vesicular phospholipid gel-based depot formulations for pharmaceutical proteins: Development and in vitro evaluation. J. Control. Release.

[8] Dai C., Wang B., Zhao H. (2005). Microencapsulation peptide and protein drugs delivery system. Colloids Surfaces B.

[9] Sinha V.R., Trehan A. (2003). Biodegradable microspheres for protein delivery. J. Control. Release.

[10] Del Gaudio C., Crognale V., Serino G., Galloni P., Audenino A., Ribatti D., Morbiducci U. (2017). Natural polymeric microspheres for modulated drug delivery. Mater. Sci. Eng. C Mater. Biol. Appl..

[11] Gomez-Gaete C., Retamal M., Chavez C., Bustos P., Godoy R., Torres-Vergara P. (2017). Development, characterization and in vitro evaluation of biodegradable rhein-loaded microparticles for treatment of osteoarthritis. Eur. J. Pharm. Sci..

[12] Wei X., Gong C., Gou M., Fu S., Guo Q., Shi S., Luo F., Guo G., Qiu L., Qian Z. (2009). Biodegradable poly(epsilon-caprolactone)-poly(ethylene glycol) copolymers as drug delivery system. Int. Pharm..

[13] Paciello A., Amalfitano G., Garziano A., Urciuolo F., Netti P.A. (2016). Hemoglobin-Conjugated Gelatin Microsphere as a Smart Oxygen Releasing Biomaterial. Adv. Healthc. Mater..

[14] Huang Z., Chen X., Fu H., Wen X., Ma C., Zhang J., Wu C., Huang Y., Pan X., Wu C. (2017). Formation Mechanism and In Vitro Evaluation of Risperidone-Containing PLGA Microspheres Fabricated by Ultrafine Particle Processing System. J. Pharm. Sci..

[15] Siafaka P.I., Barmpalexis P., Lazaridou M., Papageorgiou G.Z., Koutris E., Karavas E., Kostoglou M., Bikiaris D.N. (2015). Controlled release formulations of risperidone antipsychotic drug in novel aliphatic polyester carriers: Data analysis and modelling. Eur. J. Pharm. Biopharm..

[16] Hoffman A.S. (2008). The origins and evolution of “controlled” drug delivery systems. J. Control. Release.

[17] Wu F., Jin T. (2008). Polymer-Based Sustained-Release Dosage Forms for Protein Drugs, Challenges, and Recent Advances. AAPS PharmSciTech.

[18] Mitragotri S., Burke P.A., Langer R. (2014). Overcoming the challenges in administering biopharmaceuticals: Formulation and delivery strategies. Nat. Rev. Drug Discov..

[19] Mao S., Guo C., Shi Y., Li L.C. (2012). Recent advances in polymeric microspheres for parenteral drug delivery—Part 1. Expert Opin. Drug Deliv..

[20] Gasmi H., Siepmann F., Hamoudi M.C., Danede F., Verin J., Willart J.F., Siepmann J. (2016). Towards a better understanding of the different release phases from PLGA microparticles: Dexamethasone-loaded systems. Int. Pharm..

[21] Casalini T., Rossi F., Lazzari S., Perale G., Masi M. (2014). Mathematical modeling of PLGA microparticles: From polymer degradation to drug release. Mol. Pharm..

[22] Park E.J., Amatya S., Kim M.S., Park J.H., Seol E., Lee H., Shin Y.H., Na D.H. (2013). Long-acting injectable formulations of antipsychotic drugs for the treatment of schizophrenia. Arch. Pharm. Res..

[23] Janssen P.A., Niemegeers C.J., Awouters F., Schellekens K.H., Megens A.A., Meert T.F. (1988). Pharmacology of risperidone (R 64 766), a new antipsychotic with serotonin-S2 and dopamine-D2 antagonistic properties. J. Pharmacol. Exp. Ther..

[24] Cheng F., Jones P.B. (2013). Drug treatments for schizophrenia: Pragmatism in trial design shows lack of progress in drug design. Epidemiol. Psychiatr. Sci..

[25] Ereshefsky L., Mascarenas C.A. (2003). Comparison of the effects of different routes of antipsychotic administration on pharmacokinetics and pharmacodynamics. J. Clin. Psychiatry.

[26] Yerragunta B., Jogala S., Chinnala K.M., Aukunuru J. (2015). Development of a novel 3-month drug releasing risperidone microspheres. J. Pharm. Bioallied Sci..

[27] Su Z., Sun F., Shi Y., Jiang C., Meng Q., Teng L., Li Y. (2009). Effects of formulation parameters on encapsulation efficiency and release behavior of risperidone poly(d,l-lactide-co-glycolide) microsphere. Chem. Pharm. Bull..

[28] Su Z.X., Shi Y.N., Teng L.S., Li X., Wang L.X., Meng Q.F., Teng L.R., Li Y.X. (2011). Biodegradable poly(d,l-lactide-co-glycolide) (PLGA) microspheres for sustained release of risperidone: Zero-order release formulation. Pharm. Dev. Technol..

[29] Hu Z., Liu Y., Yuan W., Wu F., Su J., Jin T. (2011). Effect of bases with different solubility on the release behavior of risperidone loaded PLGA microspheres. Colloids Surfaces B.

[30] Karavelidis V., Giliopoulos D., Karavas E., Bikiaris D. (2010). Nanoencapsulation of a water soluble drug in biocompatible polyesters. Effect of polyesters melting point and glass transition temperature on drug release behavior. Eur. J. Pharm. Sci..

[31] Karavelidis V., Bikiaris D., Avgoustakis K. (2015). New thermosensitive nanoparticles prepared by biocompatible pegylated aliphatic polyester block copolymers for local cancer treatment. J. Pharm. Pharmacol..

[32] Solomon O.F., Ciutǎ I.Z. (1962). Détermination de la viscosité intrinsèque de solutions de polymères par une simple détermination de la viscosité. J. Appl. Polym. Sci..

[33] Barmpalexis P., Kachrimanis K., Malamataris S. (2018). Statistical moments in modelling of swelling, erosion and drug release of hydrophilic matrix-tablets. Int. Pharm..

[34] Nanaki S.G., Pantopoulos K., Bikiaris D.N. (2011). Synthesis of biocompatible poly(varepsilon-caprolactone)-block-poly(propylene adipate) copolymers appropriate for drug nanoencapsulation in the form of core-shell nanoparticles. Int. J. Nanomed..

[35] Beslikas T., Gigis I., Goulios V., Christoforides J., Papageorgiou G.Z., Bikiaris D.N. (2011). Crystallization study and comparative in vitro-in vivo hydrolysis of PLA reinforcement ligament. Int. J. Mol. Sci..

[36] Papageorgiou G., Beslikas T., Gigis J., Christoforides J., Bikiaris D.N. (2010). Crystallization and enzymatic hydrolysis of PLA grade for orthopedics. Adv. Polym. Technol..

[37] Bikiaris D.N., Nianias N.P., Karagiannidou E.G., Docoslis A. (2012). Effect of different nanoparticles on the properties and enzymatic hydrolysis mechanism of aliphatic polyesters. Polym. Degrad. Stab..

[38] Qiu Z., Ikehara T., Nishi T. (2003). Poly(hydroxybutyrate)/poly(butylene succinate) blends: Miscibility and nonisothermal crystallization. Polymer.

[39] Gan Z., Abe H., Kurokawa H., Doi Y. (2001). Solid-State Microstructures, Thermal Properties, and Crystallization of Biodegradable Poly(butylene succinate) (PBS) and Its Copolyesters. Biomacromolecules.

[40] Daniel J.S.P., Veronez I.P., Rodrigues L.L., Trevisan M.G., Garcia J.S. (2013). Risperidone—Solid-state characterization and pharmaceutical compatibility using thermal and non-thermal techniques. Thermochim. Acta.

[41] Pappa C., Nanaki S., Giliopoulos D., Triantafyllidis K., Kostoglou M., Avgeropoulos A., Bikiaris D. (2018). Nanostructured Composites of Sodium Montmorillonite Clay and PEO Used in Dissolution Improvement of Aprepitant Drug by Melt Mixing. Appl. Sci..

[42] Nanaki S., Tseklima M., Terzopoulou Z., Nerantzaki M., Giliopoulos D.J., Triantafyllidis K., Kostoglou M., Bikiaris D.N. (2017). Use of mesoporous cellular foam (MCF) in preparation of polymeric microspheres for long acting injectable release formulations of paliperidone antipsychotic drug. Eur. J. Pharm. Biopharm..

[43] Nanaki S., Siafaka P.I., Zachariadou D., Nerantzaki M., Giliopoulos D.J., Triantafyllidis K.S., Kostoglou M., Nikolakaki E., Bikiaris D.N. (2017). PLGA/SBA-15 mesoporous silica composite microparticles loaded with paclitaxel for local chemotherapy. Eur. J. Pharm. Sci..

[44] Iordanskii A.L., Zaikov G.E., Berlin A.A. (2015). Diffusion kinetics of hydrolysis of biodegradable polymers. Weight loss and control of the release of low molecular weight substances. Polym. Sci. Ser. D.

[45] Tien C. (1994). Adsorption Calculations and Modeling.

[46] Korsmeyer R.W., Gurny R., Doelker E., Buri P., Peppas N.A. (1983). Mechanisms of solute release from porous hydrophilic polymers. Int. Pharm..

[47] Crank J. (1979). The Mathematics of Diffusion.

[48] Georgiadis M.C., Kostoglou M. (2001). On the optimization of drug release from multi-laminated polymer matrix devices. J. Control. Release.

